# Inhibition of Autophagy via Activation of PI3K/Akt Pathway Contributes to the Protection of Ginsenoside Rb1 against Neuronal Death Caused by Ischemic Insults

**DOI:** 10.3390/ijms150915426

**Published:** 2014-09-01

**Authors:** Tianfei Luo, Guiying Liu, Hongxi Ma, Bin Lu, Haiyang Xu, Yujing Wang, Jiang Wu, Pengfei Ge, Jianmin Liang

**Affiliations:** 1 Department of Neurology, First Hospital of Jilin University, Changchun 130021, China; E-Mails: luotianfei2010@126.com (T.L.); drwujiang2010@163.com (J.W.); 2 Department of Pediatrics, Anzhen Hospital of Capital University of Medical Sciences, Beijing 10029, China; E-Mail: liugvying@126.com; 3 Department of Pathology, First Hospital of Jilin University, Changchun 130021, China; E-Mail: mahongxi1969@163.com; 4 Department of Neurosurgery, First Hospital of Jilin University, Changchun 130021, China; E-Mails: Lubin03216@163.com (B.L.); xuhaiyang76@163.com (H.X.); 5 Department of Pediatrics, First Hospital of Jilin University, Changchun 130021, China; E-Mail: yujingwang2012@126.com

**Keywords:** autophagy, OGD, transient global ischemia, Ginsenoside Rb1, PI3K/Akt

## Abstract

Lethal autophagy is a pathway leading to neuronal death caused by transient global ischemia. In this study, we examined the effect of Ginsenoside Rb1 (GRb1) on ischemia/reperfusion-induced autophagic neuronal death and investigated the role of PI3K/Akt. Ischemic neuronal death *in vitro* was induced by using oxygen glucose deprivation (OGD) in SH-SY5Y cells, and transient global ischemia was produced by using two vessels occlusion in rats. Cellular viability of SH-SY5Y cells was assessed by MTT assay, and CA1 neuronal death was evaluated by Hematoxylin-eosin staining. Autophagic vacuoles were detected by using both fluorescent microscopy in combination with acridine orange (AO) and Monodansylcadaverine (MDC) staining and transmission electronic microscopy. Protein levels of LC3II, Beclin1, total Akt and phosphor-Akt at Ser473 were examined by western blotting analysis. GRb1 inhibited both OGD and transient ischemia-induced neuronal death and mitigated OGD-induced autophagic vacuoles in SH-SY5Y cells. By contrast, PI3K inhibitor LY294002 counteracted the protection of GRb1 against neuronal death caused by either OGD or transient ischemia. LY294002 not only mitigated the up-regulated protein level of phosphor Akt at Ser473 caused by GRb1, but also reversed the inhibitory effect of GRb1 on OGD and transient ischemia-induced elevation in protein levels of LC3II and Beclin1.

## 1. Introduction

Ischemic stroke due to lack of cerebral blood supply is one of the most common causes leading to death or disability in adults worldwide [1]. Transient global brain ischemia arising during cardiac arrest, cardiac surgery or induced experimentally in animals via bilateral carotid artery occlusion, causes highly selective, delayed neuronal death [2]. Although multiple factors such as oxidative stress, endoplasmic reticulum stress, and calcium overload have been identified to participate in the process of ischemic neuronal death [3], autophagy is also found to play an important role in regulating neuronal death not only induced by trauma and hypoxia [4,5], but also by ischemia/reperfusion [6]. Autophagy is an evolutionarily conserved and highly regulated homeostatic process by which cytoplasmic macromolecules and organelles are degraded for removal or turnover through a lysosomal system [7]. Physiologically, autophagy at basal level maintains a stable intracellular environment. Defects in autophagy are found to be associated with neuronal loss in neurodegenerative diseases, in which abnormal proteins and damaged organelles could not be cleared from neurons [7]. By contrast, over-activated autophagy triggers non-apoptotic programmed cell death (autophagic cell death) through excessive self-digestion and degradation of essential cellular constituents [8]. In brain and spine, activated autophagy caused by transient ischemia promoted neuronal damage [9,10]. Also, autophagy aggravated cell death is induced by ischemia and reperfusion in heart and liver [11,12]. Thus, these previous studies revealed that autophagy is a common pathway leading to cell death in different organs stressed by transient ischemia.

Ginsenoside Rb1 (GRb1) is a main ingredient isolated from the root of Panax ginseng C.A. Meyer that has been used as a very important component of Chinese prescriptions for thousands of years [3]. GRb1 has been studied extensively and found to have multiple biological functions, including anti-inflammation, anti-apoptosis and induction of neurogenesis [13,14,15]. Particularly, GRb1 was demonstrated to be a potent agent that could inhibit ischemia/reperfusion-induced cellular death in heart, liver and brain [16,17,18]. As a protectant against neuronal injury, GRb1 is found to inhibit the expression of autophagy-related proteins such as Beclin1 and LC3 induced by either focal ischemia/reperfusion [19] or neurotoxic glutamate [20], though its underlying mechanism is still elusive. The PI3K/Akt pathway is a central mediator in signal transduction pathways involved in cell growth, cell survival, and metabolism. Accumulating evidences show that it plays a pivotal role in defense against various neuro-damaging insults [21]. Phosphorylated Akt, the activated form of Akt, could activate another intracellular serine/threonine kinase mammalian target of rapamycin (mTOR), a major intracellular repressor of autophagy. Recent studies suggest that enhanced activation of Akt would lead to inhibition of lethal autophagy [22]. Thus, we speculate that activation of the PI3K/Akt pathway might contribute to the inhibitory effect of GRb1 on excessive autophagy caused by ischemia/reperfusion. In this study, we used *in vitro* and *in vivo* experimental models of cerebral ischemic injury to investigate the role of the PI3k/Akt pathway in the protection of GRb1 against lethal autophagy.

## 2. Results and Discussion

### 2.1. Ginsenoside Rb1 (GRb1) Inhibits Neuronal Death Induced by Oxygen Glucose Deprivation (OGD)

**Figure 1:**
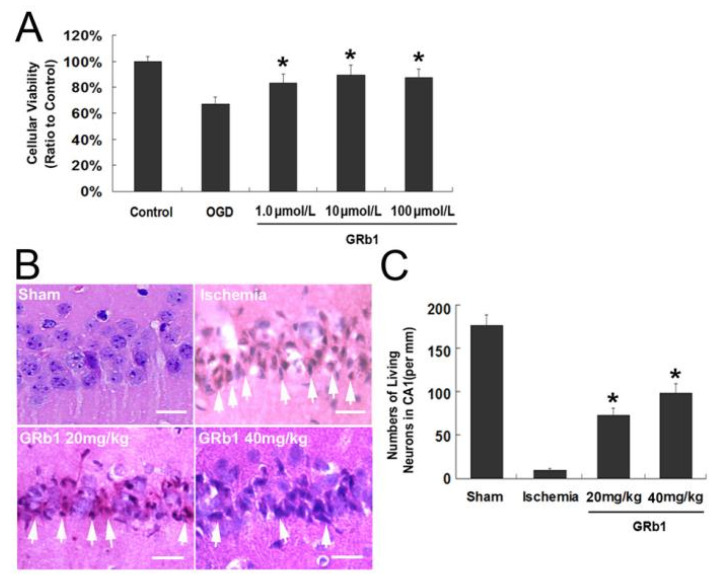
Protective effect of Ginsenoside Rb1 (GRb1) on both oxygen glucose deprivation (OGD)-induced cell death in SH-SY5Y cells and neuronal death in CA1 region caused by transient global ischemia. (**A**) MTT assay of cellular viability of SH-SY5Y cells. The reduction in the cellular viability at recovery 24 h following OGD treatment was counteracted by administration of GRb1; (**B**) Histological images of the CA1 neurons stained with hematoxylin and eosin 40×. The dead neurons indicated by arrows presented condensed polyglonal nuclei and pink cytosols; (**C**) Statistical analysis of living neurons in the CA1 region. Scale bar = 20 µm. * *p* < 0.01 versus OGD group. Each experiment was performed four times.

To investigate the protection of GRb1 against neuronal death, we compared cellular viability at recovery 24 h between the groups treated with oxygen glucose deprivation (OGD) and treated with GRb1. As Figure 1A showed, the percentage of cellular viability was 66.82% ± 5.36% in the OGD group, which was improved by pretreatment with GRb1 to 83.25% ± 5.72% (*p* < 0.01 *versus* OGD group) and 89.35% ± 6.16% (*p* < 0.01 *versus* OGD group) at concentration of 1.0 and 10 µmol/L, respectively. Despite that 100 µmol/L GRb1 showed protective effects on OGD-induced death in SH-SY5Y cells, no significant difference could be found in cellular viability when compared with the group treated by 10 µmol/L GRb1. Therefore, we used the concentrations of 1.0 and 10 µmol/L to investigate the mechanism underlying the protection of GRb1 on OGD-induced cell death.

### 2.2. GRb1 Inhibits OGD-Induced Autophagy in SH-SY5Y Cells

**Figure 2:**
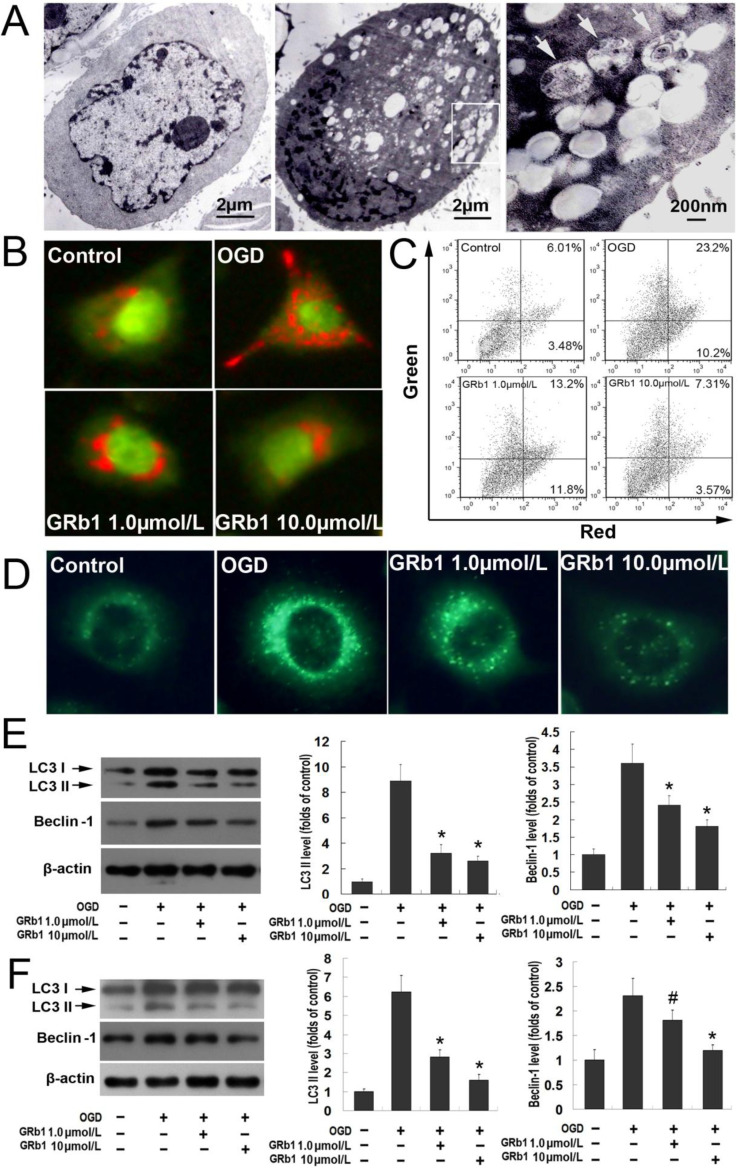
GRb1 inhibited OGD-induced autophagic death in SH-SY5Y cells. (**A**) Images acquired by transmission electron microscope. Compared with control group, many autophagic vacuoles formed in the cytoplasm of SH-SY5Y cells stressed by OGD at recovery 24 h. Vacuoles containing organelles were indicated by arrows; (**B**) Images of acridine orange (AO) staining under fluorescence microscope. Many red spots formed in the cytoplasm of SH-SY5Y cells, which were attenuated by administration of GRb1 either at concentration of 1.0 or 10 µmol/L; (**C**) Flow cytometric assay of AO staining; (**D**) Images of Monodansylcadaverine (MDC) staining. The fluo-density in the OGD group was higher than that in the control group, but it was mitigated effectively by 1.0 and 10 µmol/L GRb1; (**E**) Western blotting analysis of the expression of autophagy hallmark proteins Beclin1 and LC3II at 12 h recovery; (**F**) Western blotting analysis of the expression of Beclin1 and LC3II at recovery 24 h. The protein levels of Beclin1 and LC3II increased significantly at 12 and 24 h recovery following OGD treatments, when compared with those in the control group. However, they were both suppressed by treatment with GRb1. Moreover, the inhibitory effect of GRb1 at 10 µmol/L was stronger than that at 1.0 µmol/L. * *p* < 0.01 *versus* OGD group; ^#^ *p* < 0.05 *versus* OGD group. Each experiment was performed four times.

Autophagy was reported to contribute to programmed neuronal death caused by OGD [23]. In this study, it was revealed by transmission electron microscopy that many autophagic vacuoles formed in the cytoplasm of SH-SY5Y cells at recovery 24 h after OGD, but not in normal SH-SY5Y cells (Figure 2A). AO (acridine orange) and MDC (Monodansylcadaverine) are fluorescent substances, which are often used to detect the occurrence of autophagy. As shown in Figure 2B,D, when compared with the control group, OGD induced far more red spots and stronger punctate fluorescence of MDC at 24 h recovery in the cytoplasm of SH-SY5Y cells. However, these alterations were suppressed by pretreatment with GRb1, especially at higher concentration. Moreover, acridine orange vital staining was quantified by using FACS analysis. We found that the increment of acridine orange-positive acidic vesicular organelles (AVO) in the OGD group was inhibited by administration of GRb1 prior to OGD either at the concentration of 1.0 or 10 µmol/L. Although western blotting analysis proved that the protein levels of autophagic hallmark proteins Beclin1 and LC3II in the OGD group at recovery of 12 and 24 h were both higher than those in the control group (*p* < 0.01), either 1.0 or 10 µmol/L GRb1 markedly attenuated OGD-induced elevation in the protein levels of Beclin1 and LC3II. Thus, these data indicated that GRb1 could inhibit OGD-induced lethal autophagy.

### 2.3. GRb1 Inhibits OGD-Induced Autophagic Death via Activation of PI3K/Akt Pathway

**Figure 3:**
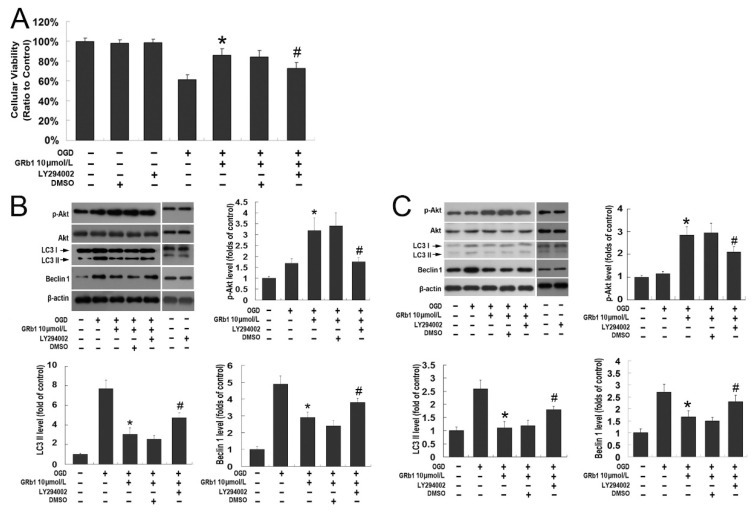
The PI3K/Akt pathway contributed to the protection of ginsenoside Rb1 against lethal autophagy caused by OGD. (**A**) MTT assay of cellular viability in SH-SY5Y cells. Although 10.0 µmol/L GRb1 significantly inhibited OGD-induced cell death in SH-SY5Y cells at 24 h recovery, pretreatment with PI3K inhibitor LY294002 prior to GRb1 reversed the protection of GRb1. DMSO, the solvent of LY294002, did not show any effect on the protection of GRb1; (**B**) Images of Western blotting and statistics of optical densities of immunoblots at 12 h recovery; (**C**) Images of Western blotting and statistics of optical densities of immunoblots at 24 h recovery. The elevated protein level of phosphor-Akt at Ser473 and the reduced expression of Beclin1 and LC3II caused by GRb1 were all counteracted by pretreatment with PI3K inhibitor LY294002 either at 12 or 24 h recovery. * *p* < 0.01 versus OGD group; # *p* < 0.01 versus GRb1 group. Each experiment was performed four times.

PI3K and its downstream-regulated protein, Akt, are known to protect neuronal damage caused by ischemia/reperfusion [24]. Although 10 µmol/L PI3K inhibitor LY294002 and its solvent DMSO did not affect the viability of SH-SY5Y cells when compared with the control group, pretreatment with LY194002 counteracted the protection of GRb1 against OGD-induced cell death in SH-SY5Y cells (Figure 3A). At 24 h recovery, the cellular viability was 85.79% ± 6.57% in the GRb1 group, but it was reduced to 72.53% ± 6.02% when LY294002 was administrated prior to GRb1 treatment (*p* < 0.01). As shown in Figure 3B,C, no significant difference could be found in the protein levels of phosphor-Akt at Ser 473 (the activated form of Akt), Beclin1 and LC3II between control SH-SY5Y cells and cells treated with LY294002 alone. By contrast, western blotting analysis showed that GRb1-induced elevation in the protein level of phosphor-Akt at Ser473 was inhibited by LY294002 either at 12 or 24 h recovery, despite that no significant changes could be found in total Akt level between each experimental group (Figure 3B,C). Moreover, LY294002 reversed the inhibitory effect of GRb1 on the elevated protein levels of Beclin1 and LC3II induced by OGD. Thus, these data suggest that the protection of GRb1 against OGD-induced autophagic cell death is associated with activation of the PI3K/Akt pathway.

### 2.4. GRb1 Inhibits Lethal Autophagy Caused by Transient Cerebral Ischemia

**Figure 4:**
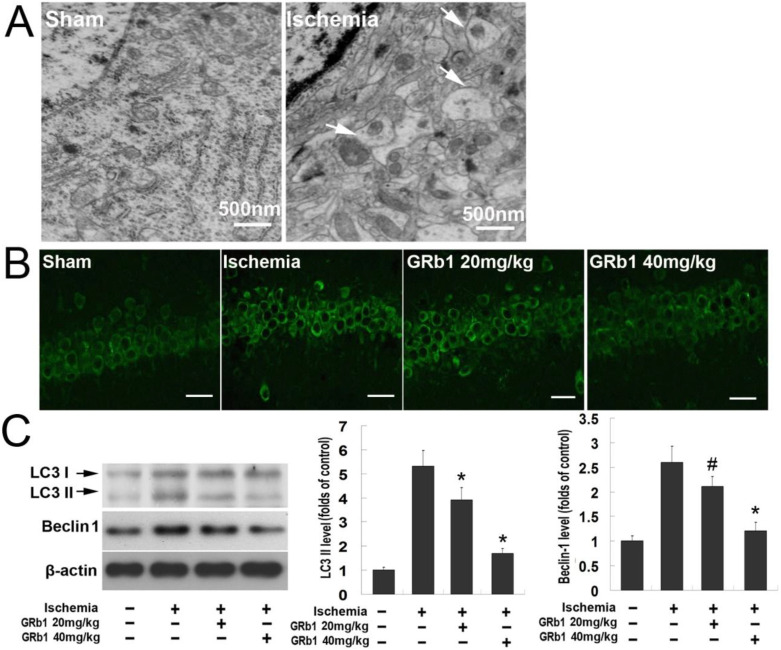
GRb1 inhibited autophagy in CA1 neurons caused by transient global ischemia. (**A**) Images of CA1 neurons under transmission electron microscope. Compared with sham groups, many vacuoles contained mitochondria formed in the cytoplasm in CA1 neurons (indicated by arrows); (**B**) Laser scanning confocal microscopic images of CA1 neurons stained immunochemically with anti-LC3 antibody. Elevated expression of LC3 at reperfusion 24 h following cerebral ischemia was mitigated effectively by pretreatment with GRb1 at doses of 20 and 40 mg/kg. Scale bar = 30 µm; (**C**) Images of western blotting and statistics of optical densities of immunoblots. * *p* < 0.01 *versus* OGD group; ^#^ *p* < 0.05 *versus* OGD group. Each experiment was performed four times.

On the basis of previous reports [25], we used the 20 and 40 mg/kg doses of GRb1 to investigate the effect of GRb1 on neuronal injury caused by transient ischemia. As shown in Figure 1B, when compared with the CA1 neurons in the sham group, almost all the neurons in the CA1 region presented condensed polyglonal nuclei and pink cytosols at reperfusion 72 h following cerebral ischemia, which was consistent with the pathological features of dead neurons described previously [14]. By contrast, the dead neurons decreased markedly in the groups treated with GRb1. Statistical results showed that the living neurons were 9.29 ± 1.91 in ischemia group. By contrast, GRb1 treatment increased the living neurons to 72.86 ± 8.32 and 98.67 ± 10.22 at doses of 20 and 40 mg/kg, respectively (Figure 1C). Figure 4A shows that transmission electron microscopy revealed many autophagic vesicles formed in the cytoplasm in CA1 neurons at reperfusion at 24 h when compared with the sham group. Consistently, confocal microscopy showed the protein level of autophagy hallmark protein LC3 in CA1 neurons of ischemia group was also significantly higher than that in control group (Figure 4B). Moreover, western blotting analysis proved as well that not only the protein level of LC3II, but also Beclin1 was upregulated at reperfusion at 24 h. By contrast, pretreatment with GRb1 at lower or higher doses markedly attenuated the increased protein levels of Beclin1 and LC3II caused by ischemia and reperfusion (Figure 4B,C). Moreover, the inhibitory effect of 40 mg/kg GRb1 on these two proteins was stronger than that of 20 mg/kg GRb1; we thus used 40 mg/kg in the subsequent experiments.

### 2.5. GRb1 Attenuated Transient Ischemia-Induced Autophagic Neuronal Death via Activation of the PI3K/Akt Pathway

**Figure 5:**
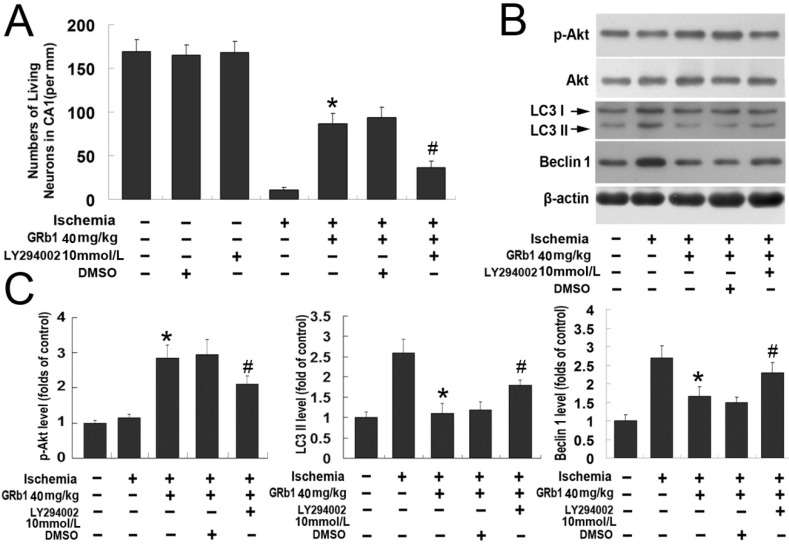
PI3K/Akt pathway contributed to the protection of ginsenoside Rb1 on neuronal death caused by transient global ischemia. (**A**) Statistical analysis of living neurons in the CA1 region. PI3K inhibitor LY294002 suppressed the protection of GRb1 at 40 mg/kg against neuronal death caused by transient ischemia, but its solvent DMSO did not; (**B**) Images of western blotting; (**C**) Statistics of optical densities of immunoblots. GRb1-induced up-regulated expression of phosphor Akt at Ser473 and down-regulated level of LC3II were all attenuated by pretreatment with LY294002. * *p* < 0.01 *versus* OGD group; ^#^ *p* < 0.01 *versus* GRb1 group. Each experiment was performed four times.

Similar to *in vitro* study, we examined the role of PI3K/Akt in the modulation of the neuroprotection of GRb1 *in vivo*. As shown in Figure 5A, intracerebroventricular injection of LY294002 and its solvent DMSO did not induce neuronal death in the CA1 region when compared with the control group, but LY294002 significantly mitigated the pro-survival effect of GRb1 on neuronal death caused by transient ischemia. The living neurons in the CA1 region was 36.39 ± 6.82 in the group treated with LY294002 followed by GRb1, which was lower than 93.86 ± 11.43 in the group treated with GRb1 alone and 102.33 ± 10.12 in the group treated with DMSO before GRb1 administration (*p* < 0.01), indicating LY294002 counteracted the protection of GRb1 against neuronal death caused by transient ischemia and reperfusion. Moreover, as shown in Figure 5B,C, western blotting analysis revealed that the up-regulated level of phosphor-Akt at Ser473 at 24 h reperfusion due to administration of GRb1 was attenuated significantly by pretreatment with LY294002, although the total Akt level in each experimental group did not obviously change. By contrast, the protein levels of Beclin1 and LC3II in the LY294002 group were significantly higher than those in the GRb1 group. Moreover, no significant changes could be found in the proteins levels of phosphorylated Akt at Ser473, Beclin1 and LC3II between the DMSO group and the GRb1 group. Therefore, the *in vivo* study also showed that activation of the PI3K/Akt pathway contributes to the protection of GRb1 against autophagic neuronal death induced by ischemia and reperfusion.

### 2.6. Discussion

In this study, we found that the protection of GRb1 against neuronal death induced by OGD or transient global ischemia is associated with suppression of excessive autophagy. Moreover, the inhibitory effect of GRb1 on autophagy is via activation of the PI3K/Akt pathway.

Autophagic cell death, as well as apoptosis, is a type of programmed cell death involved in the process of delayed neuronal death caused by transient ischemia [26,27,28]. Although induction of autophagy is found to exert protection on neuronal injury in neurodegenerative diseases [29], overly activated autophagy would make neurons enter into the autophagic cell death pathway [30]. Under condition of global transient ischemia, autophagy activation was reported to parallel neuronal death in the CA1 region of rat hippocampus, and autophagy inhibitors, such as 3-Methyladenine (3-MA) could reverse neuronal damage [31]. Similar results were found in PC12 cells stressed by oxygen glucose deprivation, an *in vitro* model mimicking ischemia and reperfusion [23]. Moreover, inhibition of autophagy was proved to be responsible for the protection of ischemic preconditioning against brain damage caused by focal ischemia in rats [32]. Thus, these studies indicate that excessive autophagy participates in the process of delayed neuronal death caused by transient ischemia.

Previously, we and other researchers have demonstrated that anti-apoptosis contributes to the protection of GRb1 against neuronal death by using *in vitro* and *in vivo* models of ischemic injury [3,18]. In this study, our data showed that the protection of GRb1 against neuronal death induced by OGD or transient global ischemia is also associated with suppression of excessive autophagy. The protection of GRb1 at higher concentration against neuronal damage is better than GRb1 at lower concentration, concomitant with stronger inhibition of autophagy. With increased GRb1 doses, OGD or transient ischemia-induced expressions of autophagy hallmark proteins Beclin1 and LC3II were reduced more significantly. LC3, the microtubule-associated protein 1 light chain 3, exists in cytosolic form (LC3-I) and membrane bound form (LC3-II). Increase in LC3-II is closely correlated with the extent of autophagosome formation. Beclin 1 is essential for the recruitment of other autophagic proteins during the expansion of pre-autophagosomal membranes [33]. The inhibitory effect of GRb1 on excessive autophagy has been also examined by using other neuronal injury models. Chen *et al.* found that GRb1 exerted protection against glutamate-induced neurotoxicity in cultured cortical neurons via inhibition of autophagy [34]. By using focal ischemia and reperfusion models in rats, Lu *et al.* found that GRb1 not only ameliorated cerebral infarction in rats, but also inhibited the expression of autophagy-related proteins LC3 and Beclin-1 [19]. Thus, these studies indicate GRb1 could attenuate neuronal death by inhibition of excessive autophagy, as well as by inhibition of apoptosis.

Though it was unclear how GRb1 regulates lethal autophagy, it had been found that the activated PI3k/Akt pathway participates in the regulation of both apoptosis and autophagy [34,35]. Thus, we investigated the effect of GRb1 on the activation of the PI3k/Akt pathway. Ouyang *et al.* found that the level of phosphor-Akt at Ser473 in CA1 neurons increased significantly at reperfusion 30 min and 4 h following global ischemia, but it was then reduced markedly at reperfusion at 24 h and until neuronal death [36]. Similarly, under the condition of cerebral focal ischemia, phosphor-Akt at Ser473 increased at 1 and 4 h after ischemia, but decreased significantly at 24 h after reperfusion [37]. Thus, in the present study, we compared the level of phosphorylated Akt at reperfusion at 24 h between the groups treated with and without GRb1. We found GRb1 significantly improved the level of phosphor-Akt at 473. Additionally, PI3k inhibitor LY294002 not only mitigated GRb1-induced elevation of the level of phosphorylated Akt, but also reversed the protection of GRb1 against neuronal death caused by either OGD or transient ischemia. Similarly, the regulatory effects of GRb1 on the activation of PI3k/Akt induced by transient ischemia was examined by other researchers; Guo *et al.* found that PI3K/Akt activation contributed to the protection of GRb1 postconditioning against hepatocytes injury caused by ischemia-reperfusion [17] and Wang *et al.* reported that GRb1 preconditioning protected against myocardial infarction after regional ischemia and reperfusion by activation of PI3k/Akt [38]. In this study, we firstly demonstrated that GRb1 protects neuronal injury induced by ischemic insults via activation of the PI3k/Akt pathway. Therefore, our study provides evidence that the protective effect of GRb1 against neuronal damage also relies on the activation of the PI3K/Akt pathway, which agrees with the results of previous studies.

Moreover, we found that the inhibited expressional levels of Beclin-1 and LC3II due to GRb1 treatment were reversed when Akt phosphorylation was suppressed by PI3k inhibitor LY294002. Similarly, Yasuda *et al.* reported that Mdm20 stimulates polyQ aggregation via inhibiting autophagy through phosphorylation of Akt at serine 473 [39]. Cheng *et al.* reported that Akt suppressed retrograde degeneration of dopaminergic axons by inhibition of autophagy [40]. Currently, accumulating evidence has suggested that the PI3K/Akt pathway plays an important role in the regulation of autophagic neuronal death. Zheng *et al.* reported that melatonin inhibited ischemia/reperfusion induced autophagy via augmenting the activation of the PI3K/Akt pathway [6]. Yansong *et al.* found that PI3K/Akt participated in the protection of IGF-1 against NMDA-induced excitotoxicity in cultured hippocampal neurons via inhibition of autophagy [21]. By contrast, it was reported that inhibition of the PI3k/Akt pathway led to autophagic death in cardiomyocytes [22]. Except for activation of the PI3k/Akt pathway, GRb1 could counteract neuronal damage via other mechanisms, including inhibition of oxidative stress [41], mitigation of endoplasmic reticulum stress [42] and induction of DNA repair [43].

## 3. Experimental Section

### 3.1. Reagents

Ginsenoside Rb1 (purity > 98%) was obtained from the National Institute for the Control of Pharmaceutical and Biological Products (Beijing, China). It was dissolved in physiological saline (0.9% NaCl) at a concentration of 10 mmol/L as stock solutions. It was diluted with cell culture medium for *in vitro* study, and diluted with physiological saline *in vivo* study.

### 3.2. Cell Culture and Treatment

Human SH-SY5Y cells were obtained from Shanghai Institute of Cell Biology, Chinese Academy of Sciences (Shanghai, China). Cells were cultured in DMEM supplemented with 10% fetal bovine serum, 2 mmol/L glutamine (Gibco, Grand Island, NY, USA), penicillin (100 U/mL) and streptomycin (100 μg/mL), and maintained at 37 °C and 5% CO_2_ in a humid environment.

As described previously [14], cells were placed in an anaerobic chamber and incubated for 3 h with glucose-free balanced salt solution that had been bubbled with an anaerobic gas mix (95% N_2_, 5% CO_2_) to produce lethal oxygen–glucose deprivation. OGD was terminated by replacing the exposure solution with normal DMEM culture and returning it to the incubator under normoxic conditions.

In experiment 1, cells were divided into control group (normal SH-SYSY cells), OGD group (cells were treated with 3 h OGD followed by 24 h recovery), GRb1 + OGD group (the cells were treated with GRb1 at 1.0, 10 and 100 µmol/L at 30 min prior to OGD, and continued at 24 h after recovery).

In experiment 2, cells were divided into control group, LY294002 group (the cells were with 10 µmol/L LY294002 alone), LY294002 vehicle group (the cells were treated with DMSO alone), OGD group, GRb1 + OGD group (the cells were treated with GRb1 as described in experimental 1), LY294002 vehicle + GRb1 + OGD group (the cells were treated with DMSO at 15 min before GRb1 and continued at 24 h after recovery), and LY294002 + GRb1 + OGD group (the cells were treated with 10 µmol/L LY294002 at 15 min before GRb1 and continued at 24 h after recovery).

### 3.3. Cell Viability Assay

SH-SY5Y cells were seeded at a density of 4 × 10^4^ cells per well on collagen-coated 96-well plates for 24 h. Cellular viability was assessed using an MTT assay, and the absorbance value at 570 nm was read using an automatic multi-well spectrophotometer (Bio-Rad, Richmond, CA, USA).

### 3.4. Acridine Orange (AO) and Monodansylcadaverine (MDC) Staining

SH-SY5Y cells (4 × 10^4^) were seeded on 96-well plates. Some cells were incubated in PBS with 1 μg/mL acridine orange (AO; Sigma–Aldrich Company, St. Louis, MO, USA) for 15 min at room temperature in the dark, others were incubated with 100 µmol/L MDC (Monodansylcadaverine, Sigma–Aldrich Company, St. Louis, MO, USA) solution for 1 h at 37 °C in the dark. After being washed with PBS, they were examined under an Olympus fluorescence microscope with 20× objective lens magnification. In addition, some cells stained with AO were harvested by trypsinization and analyzed by flow cytometry.

### 3.5. Animals and Ischemia Model

Adult male Wistar rats (weighing 280–300 g) supplied by Jilin University Experimental Animal Center were housed in a temperature-controlled room (22–25 °C) with free access to food and water on a 12-h light/dark cycle. All animal procedures were approved by the Ethical Committee for Animal Experiments, Jilin University, Changchun, China (ID No.: EA00127, 5 January 2012).

Brain ischemia was produced by using the 2-vessel occlusion (2VO) model as described previously [14]. In brief, after being anaesthetized, both of the common carotid arteries were exposed and encircled by loose ligatures, and catheters were inserted into the femoral artery and tail artery to allow blood sampling and arterial blood pressure recording. Brain ischemia was induced by withdrawing blood via the femoral artery catheter to produce a mean artery blood pressure of 50 mmHg, followed by clamping both carotid arteries for 15 min. At the end of the ischemia, the clamps were removed and the withdrawn blood was re-infused. The sham-operated rats were treated with all the surgical procedures without clipping the carotid arteries.

In experiment 1, the rats were divided randomly into Sham-operated group, ischemia/reperfusion group, and GRb1 group (the rats were treated with 20 and 40 mg/kg GRb1 at 15 min before ischemia, respectively).

In experiment 2, the rats were divided into sham-operated group, LY294002 group, LY294002 vehicle (DMSO) group, ischemia/reperfusion group, GRb1 + ischemia group, LY294002 vehicle (DMSO) + ginsenoside Rb1 + ischemia/reperfusion group, and LY294002 + ginsenoside Rb1 + ischemia/reperfusion group. Five hundred micro liters Ginsenoside Rb1 at each dose was administrated as a single bolus injection into the tail vein at 15 min before cerebral ischemia. LY294002 (10 μL of 10 mmol/L in 25% DMSO/PBS) and the same volume of the vehicle (25% DMSO in PBS) were intracerebroventricularly injected at 15 min before the GRb1 treatment (from bregma: anteroposterior, 0.8 mm; mediolateral, 1.5 mm; depth, 3.5 mm).

### 3.6. Brain Tissue Fixation

As described previously [14], the thorax was opened and the heart was disclosed after rats were anesthetized. PBS was perfused into the vascular system at 4 °C for 3 min, and PBS with 4% paraformaldehyde was perfused at 4 °C for another 3 min. Subsequently, brain tissue was taken out and put into PBS fixation solution containing 4% paraformaldehyde at 4 °C. Twelve hours later, the 13- and 50-µm coronal brain slices were cut by a vibrotome and the brain slices were selected for hematoxylin and eosin (HE) staining and immunohistochemistry labeling, respectively.

### 3.7. Hematoxylin and Eosin Staining

The 13-µm brain slices were mounted on slides, dried in a dark room, and immersed in distilled water for 1 min followed by dehydration in gradient ethanol solution. Then, they were put into hematoxylin solution for 15 s, Eosin solution for 10 s, redehydrated in gradient ethanol solution again, treated with dimethylbenzene and covered with coverslips. Under 40× objective lens of the light microscope, the living neurons were counted from the representative sections of the hippocampus CA1 region by an observer blinded to the treatment. Total number of viable neurons per mm was considered for expressing hippocampal CA1 neuronal density.

### 3.8. Immunohistochemical Analysis

The 50-µm brain slices were incubated with citrate buffer (pH 6.0) at 100 °C for 10 min, and blocked in the TBS solution containing 3% BSA and 0.2% TX-100 for 1 h at room temperature. Then, they were incubated with 1:200 rabbit polyclonal antibody against LC3B (Sigma–Aldrich, St. Louis, MO, USA) at 4 °C overnight. After washes with TBS solution containing 0.1% TX-100, the antibody-labeled brain slices were incubated with 1% BSA containing fluorescence labeled anti-rabbit IgG (1:200) for 1 h at room temperature. They were then mounted on slides and dried in the dark room, and observed under a laser scanning confocal microscope (Olympus, Tokyo, Japan).

### 3.9. Transmission Electron Microscopy

After being collected by centrifugation, SH-SY5Y cells were fixed in ice-cold 2.5% glutaraldehyde in PBS (pH 7.3), post-fixed 30 min in 1% OsO_4_ with 0.1% potassium ferricyanide, dehydrated through a graded series of ethanol (30%–90%) followed by dry acetone, and embedded in Epon. Semithin (300 nm) sections were cut, stained with 0.5% toluidine blue, and examined under a light microscope. Ultrathin sections (65 nm) were stained with 1% uranyl acetate and 0.1% lead citrate, and examined with a JEM2000EX transmission electron microscope (JEOL, Tokyo, Japan).

Brain sections were fixed with 4% glutaraldehyde for 1 h and then with 1% OsO_4_ for 2 h in 0.1 mol/L cacodylate buffer (pH 7.4). Then, they were stained with 1% aqueous uranyl acetate overnight, dehydrated in an ascending series of ethanols and dry acetone, and embedded in Durcopan ACM resin (Sigma–Aldrich, Steinheim, Germany). Ultrathin sections (0.1 µm) were cut, stained with 3% lead citrate, and examined with transmission electron microscope.

### 3.10. Protein Isolation

After centrifugation collection, the CA1 region tissue and SH-SY5Y cells were suspended in ice-cold lysis buffer and homogenized as described previously [3,14]. The homogenates were centrifuged at 10,000× *g* at 4 °C for 10 min to obtain the supernatants, the protein concentrations of which were determined by using the Bio-Rad protein assay kit.

### 3.11. Western Blotting

Equal protein amounts were electrophoresed on 10% sodium dodecyl sulfate-polyacrylamide gels and then transferred to PVDF membranes. The membranes were blocked with 3% BSA in TBS for 30 min and then incubated respectively overnight at 4 °C with the following primary antibodies including: rabbit polyclonal autophagy LC3 (1:1000; Sigma–Aldrich, St. Louis, MO, USA), goat polyclonal Beclin 1 (1:1000; Santa Cruz Biotechnology, Santa Cruz, CA, USA), rabbit antibody to Akt (1:1000; Cell Signaling Technology, Danvers, MA, USA), mouse antibody to p-S473-Akt (1:1000; Cell Signaling Technology) and mouse monoclonal β-actin (1:2000; Santa Cruz Biotechnology). The immunoblot membranes were then incubated with horseradish-peroxidase conjugated anti-mouse (1:2000; Cell Signaling Technology), anti-rabbit IgG (1:2000; Cell Signaling Technology), or donkey anti-goat IgG (1:5000; Santa Cruz Biotechnology) for 1 h at room temperature. The immunoreactive proteins were visualized on a Kodak X-omat LS film (Eastman Kodak Company, New Haven, CT, USA) with an enhanced chemiluminescence. Densitometry was performed with Kodak ID image analyses software (Eastman Kodak Company).

### 3.12. Statistical Analysis

All data represent at least four independent experiments and are expressed as mean ± SD. Statistical comparisons were made using one-way ANOVA. *p*-values of less than 0.05 were considered to represent statistical significance.

## 4. Conclusions

The current study demonstrates clearly that GRb1 attenuates autophagic neuronal death caused by ischemic insults. Activation of the PI3k/Akt pathway contributes to the inhibitory effect of GRb1 on excessive autophagy.
