# Correction: Luo et al. Inhibition of Autophagy via Activation of PI3K/Akt Pathway Contributes to the Protection of Ginsenoside Rb1 against Neuronal Death Caused by Ischemic Insults. *Int. J. Mol. Sci.* 2014, *15*, 15426–15442

**DOI:** 10.3390/ijms26209839

**Published:** 2025-10-10

**Authors:** Tianfei Luo, Guiying Liu, Hongxi Ma, Bin Lu, Haiyang Xu, Yujing Wang, Jiang Wu, Pengfei Ge, Jianmin Liang

**Affiliations:** 1 Department of Neurology, First Hospital of Jilin University, Changchun 130021, China; luotianfei2010@126.com (T.L.); drwujiang2010@163.com (J.W.); 2 Department of Pediatrics, Anzhen Hospital of Capital University of Medical Sciences, Beijing 10029, China; liugvying@126.com; 3 Department of Pathology, First Hospital of Jilin University, Changchun 130021, China; mahongxi1969@163.com; 4 Department of Neurosurgery, First Hospital of Jilin University, Changchun 130021, China; lubin03216@163.com (B.L.); xuhaiyang76@163.com (H.X.); 5 Department of Pediatrics, First Hospital of Jilin University, Changchun 130021, China; yujingwang2012@126.com

In the original publication [1], there are some mistakes in Figures 1B and 3B. Because of negligence, the sham image in Figure 1B has been used in our other article published much earlier [2]. In Figures 3B and 5B. LC3 I and LC3 II bands are similar. The replaced Figure 1 and Figure 3 are provided below. The authors state that the scientific conclusions are unaffected. This correction was approved by the Academic Editor. The original publication has also been updated.

## Figures and Tables

**Figure 1:**
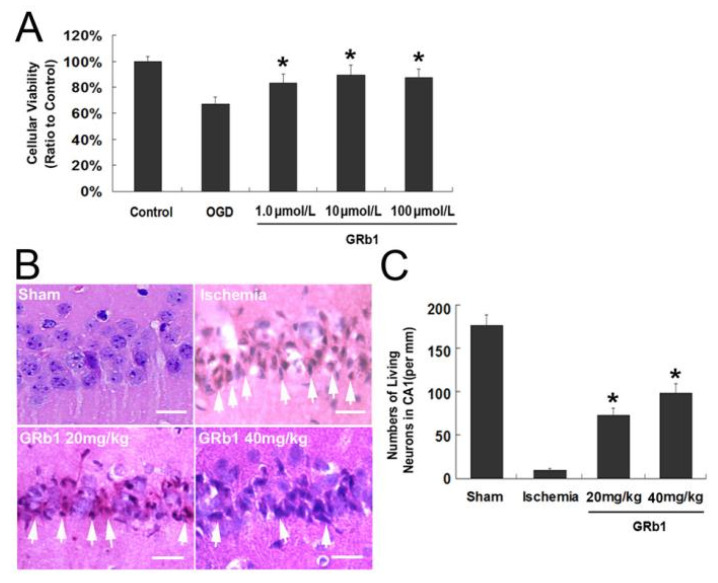
Protective effect of Ginsenoside Rb1 (GRb1) on both oxygen glucose deprivation (OGD)-induced cell death in SH-SY5Y cells and neuronal death in CA1 region caused by transient global ischemia. (**A**) MTT assay of cellular viability of SH-SY5Y cells. The reduction in the cellular viability at recovery 24 h following OGD treatment was counteracted by administration of GRb1; (**B**) Histological images of the CA1 neurons stained with hematoxylin and eosin 40×. The dead neurons indicated by arrows presented condensed polyglonal nuclei and pink cytosols; (**C**) Statistical analysis of living neurons in the CA1 region. Scale bar = 20 µm. * *p* < 0.01 versus OGD group. Each experiment was performed four times.

**Figure 3:**
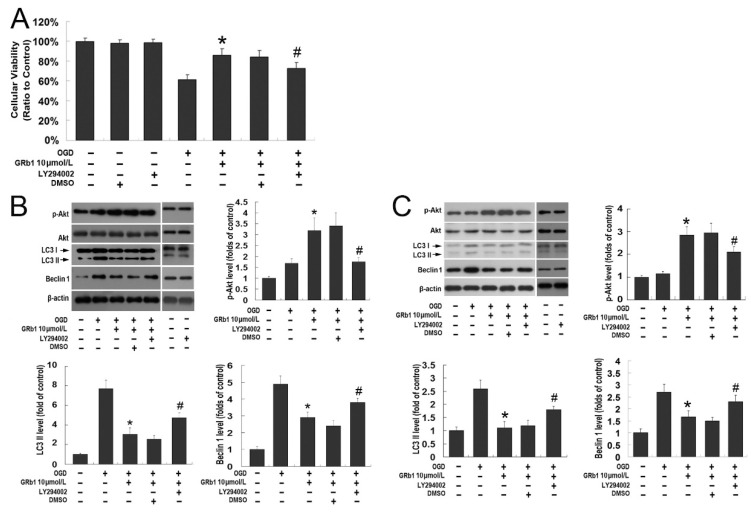
The PI3K/Akt pathway contributed to the protection of ginsenoside Rb1 against lethal autophagy caused by OGD. (**A**) MTT assay of cellular viability in SH-SY5Y cells. Although 10.0 µmol/L GRb1 significantly inhibited OGD-induced cell death in SH-SY5Y cells at 24 h recovery, pretreatment with PI3K inhibitor LY294002 prior to GRb1 reversed the protection of GRb1. DMSO, the solvent of LY294002, did not show any effect on the protection of GRb1; (**B**) Images of Western blotting and statistics of optical densities of immunoblots at 12 h recovery; (**C**) Images of Western blotting and statistics of optical densities of immunoblots at 24 h recovery. The elevated protein level of phosphor-Akt at Ser473 and the reduced expression of Beclin1 and LC3II caused by GRb1 were all counteracted by pretreatment with PI3K inhibitor LY294002 either at 12 or 24 h recovery. * *p* < 0.01 versus OGD group; # *p* < 0.01 versus GRb1 group. Each experiment was performed four times.
